# Physical activity attenuates the associations of systemic immune-inflammation index with total and cause-specific mortality among middle-aged and older populations

**DOI:** 10.1038/s41598-021-91324-x

**Published:** 2021-06-15

**Authors:** Hang Li, Xiulong Wu, Yansen Bai, Wei Wei, Guyanan Li, Ming Fu, Jiali Jie, Chenming Wang, Xin Guan, Yue Feng, Hua Meng, Mengying Li, Meian He, Xiaomin Zhang, Huan Guo

**Affiliations:** Department of Occupational and Environmental Health, Key Laboratory of Environment & Health, Ministry of Education; State Key Laboratory of Environmental Health (Incubating), School of Public Health, Tongji Medical College, Huazhong University of Science and Technology, 13 Hangkong Rd, Wuhan, 430030 Hubei China

**Keywords:** Biomarkers, Disease prevention, Public health

## Abstract

Systemic immune-inflammation index (SII) emerged as a biomarker of chronic inflammation and an independent prognostic factor for many cancers. We aimed to investigate the associations of SII level with total and cause-specific mortality risks in the general populations, and the potential modification effects of lifestyle-related factors on the above associations. In this study, we included 30,521 subjects from the Dongfeng-Tongji (DFTJ) cohort and 25,761 subjects from the National Health and Nutrition Examination Survey (NHANES) 1999–2014. Cox proportional hazards regression models were used to estimate the associations of SII with mortality from all-cause, cardiovascular diseases (CVD), cancer and other causes. In the DFTJ cohort, compared to subjects in the low SII subgroup, those within the middle and high SII subgroups had increased risks of total mortality [hazard ratio, HR (95% confidence interval, CI) = 1.12 (1.03–1.22) and 1.26 (1.16–1.36), respectively) and CVD mortality [HR (95%CI) = 1.36 (1.19–1.55) and 1.50 (1.32–1.71), respectively]; those within the high SII subgroup had a higher risk of other causes mortality [HR (95%CI) = 1.28 (1.09–1.49)]. In the NHANES 1999–2014, subjects in the high SII subgroup had higher risks of total, CVD, cancer and other causes mortality [HR (95%CI) = 1.38 (1.27–1.49), 1.33 (1.11–1.59), 1.22 (1.04–1.45) and 1.47 (1.32–1.63), respectively]. For subjects with a high level of SII, physical activity could attenuate a separate 30% and 32% risk of total and CVD mortality in the DFTJ cohort, and a separate 41% and 59% risk of total and CVD mortality in the NHANES 1999–2014. Our study suggested high SII level may increase total and CVD mortality in the general populations and physical activity exerted a beneficial effect on the above associations.

## Introduction

Systemic chronic inflammation has been considered as an important risk factor for the development of multiple chronic diseases, including cardiovascular disease (CVD), cancer, and diabetes^1^, which account for more than 50% of total deaths^2^. Since systemic chronic inflammation always developed in the absence of clinical manifestations, available and reliable systemic inflammatory markers could help identify high-risk populations and develop therapeutic strategies, which were important for reducing the burden of chronic diseases. Many observational studies have documented the associations of blood-based inflammatory markers with disease morbidity and mortality^3–5^. Elevated white blood cell (WBC) count was independently associated with increased risks of incident CVD^6^, cancer^4^, and mortality^7,8^, while the roles of WBC subtypes and other blood cells such as platelets on mortality have been also investigated individually^9–11^. As blood cells could interact with each other and then play essential roles in chronic inflammation-related diseases, the composite indices, such as neutrophil-to-lymphocyte ratio (NLR) and platelet-to-lymphocyte ratio (PLR), were considered to be potential prognostic factors for patients with CVD and cancer^12–14^, as well as markers for predicting CVD incidence and mortality in the general populations^15,16^.

Recently, the systemic immune-inflammation index (SII), calculated based on the neutrophil, lymphocyte and platelet counts in peripheral blood, was put forward to reflect the balance of systemic inflammation and immune status^17^. Previous studies implicated that an elevated level of SII was associated with poor prognosis of patients with certain malignancies, i.e., hepatocellular carcinoma^17^, colorectal cancer^18^, and pancreatic cancer^19^. Two cohort studies embedded in the Rotterdam Study found that high SII level was associated with the risks of solid cancer and dementia^20,21^. One recent cohort study of 85,154 Chinese participants reported that high SII was associated with 24% increased total mortality risk^22^, but its association with cause-specific mortality risk was not evaluated. Compelling evidence has suggested that unhealthy lifestyle factors, including smoking, alcohol drinking, physical inactivity, and being overweight or obese, contributed to about 60% of deaths^23^, and were linked to chronic inflammation status^1^, which underscored the potential modification roles of these factors on the effects of chronic inflammation.

In this study, we aimed to examine the associations of SII with total and cause-specific mortality in the general middle-aged and older populations of two large prospective cohort studies, including the Dongfeng-Tongji (DFTJ) cohort and National Health and Nutrition Examination Survey (NHANES). We also explored the modification effects of the general characteristics [age, sex, body mass index (BMI)] and common lifestyle factors (smoking, alcohol drinking and physical activity status) on the above associations.

## Results

Among the 30,521 participants in the DFTJ cohort [median (interquartile range, IQR) for age, 62.2 (56.2–68.0) years old], the median (IQR) of SII was 332.71 (236.91–467.61) × 10^9^/L. There were 3311 deaths occurring during a mean follow-up time of 8.2 years, including 1339 CVD deaths, 1081 cancer deaths, and 891 other causes deaths. Among the 25,761 participants in the NHANES 1999–2014 [median (IQR) for age, 54.5 (46.5–65.4) years old], the median (IQR) for SII was 505.14 (366.96–705.63) × 10^9^/L. There were 4828 deaths occurring during a mean follow-up time of 7.6 years, including 1093 CVD deaths, 1032 cancer deaths, and 2703 other causes deaths. As shown in Table 1, compared to survivors, the deceased participants were more likely to be males, elders, non-Hispanic Whites (only in the NHANES 1999–2014), smokers, less educated, physically inactive, and had a greater prevalence of hypertension, diabetes, CVD and cancer (all *P* < 0.05). Additionally, the deceased participants had higher neutrophil count and lower platelet count than survivors (both *P* < 0.05). The baseline SII level of the deceased participants was significantly higher than that of survivors in the NHANES 1999–2014 [median (IQR) for SII, 562.92 (392.70–824.09) × 10^9^/L vs. 497.68 (363.66–690.55) × 10^9^/L, *P* < 0.001]; however, this difference was not seen among subjects in the DFTJ cohort [median (IQR) for SII, 343.10 (229.48–487.99) × 10^9^/L vs. 331.70 (237.40–465.00) × 10^9^/L, *P* = 0.21]. The SII level was positively associated with BMI and smoking, but was inversely associated with age and physical activity in both cohorts (all *P* < 0.05, Supplementary Table S1).

**Table 1:** Baseline characteristics of study populations.

Variables	All participants	Survivors	Deaths	*P*^a^
**DFTJ cohort**				
No	30,521	27,210	3311	
Males	13,685 (44.8)	11,519 (42.3)	2166 (65.4)	< 0.001
Age (years)	62.2 (56.2–68.0)	62.0 (55.8–66.3)	70.0 (64.0–76.0)	< 0.001
BMI (kg/m^2^)	24.1 (22.0–26.4)	24.1 (22.1–26.3)	24.2 (21.9–26.6)	0.67
Smokers	9034 (29.6)	7493 (27.5)	1541 (46.5)	< 0.001
Alcohol drinkers	8730 (28.6)	7596 (27.9)	1134 (34.3)	< 0.001
*Education*				< 0.001
Below high school	18,192 (59.6)	15,842 (58.2)	2350 (71.0)	
High school	8499 (27.9)	7912 (29.1)	587 (17.7)	
College or above	3466 (11.4)	3140 (11.5)	326 (9.9)	
Missing	364 (1.2)	316 (1.2)	48 (1.5)	
Physically active	24,544 (80.4)	22,036 (81.0)	2508 (75.8)	< 0.001
*Disease history*				
Hypertension	16,678 (54.6)	14,395 (52.9)	2283 (69.0)	< 0.001
Hyperlipidemia	16,122 (52.8)	14,348 (52.7)	1774 (53.6)	0.36
Diabetes	6090 (20.0)	5027 (18.5)	1063 (32.1)	< 0.001
CVD	5226 (17.1)	4170 (15.3)	1056 (31.9)	< 0.001
Cancer	834 (2.7)	636 (2.3)	198 (6.0)	< 0.001
Neutrophils (× 10^9^/L)	3.29 (2.61–4.10)	3.23 (2.60–4.03)	3.60 (2.84–4.50)	< 0.001
Lymphocytes (× 10^9^/L)	1.83 (1.47–2.27)	1.82 (1.47–2.26)	1.87 (1.46–2.35)	0.01
Platelets (× 10^9^/L)	188 (155–225)	189 (157–226)	176 (142–216)	< 0.001
SII (× 10^9^/L) ^c^	332.71 (236.91–467.61)	331.70 (237.40–465.00)	343.10 (229.48–487.99)	0.21
**NHANES 1999–2014**				
No	**25,761**	**20,933**	**4828**	–
Males	12,640 (47.1)	9932 (46.5)	2708 (51.1)	< 0.001
Age (years)	54.5 (46.5–65.4)	52.8 (45.7–62.4)	71.7 (59.8–79.3)	< 0.001
*Race*				
Non-hispanic white	12,892 (75.0)	9956 (74.4)	2936 (78.8)	< 0.001
Non-Hispanic Black	5132 (9.8)	4265 (9.8)	867 (10.1)	
Mexican American	4276 (5.6)	3575 (5.8)	701 (4.0)	
Other	3461 (9.6)	3137 (10.0)	324 (7.1)	
BMI (kg/m^2^)	28.0 (24.6–32.2)	28.1 (24.7–32.3)	27.4 (24.0–31.6)	< 0.001
Smokers	12,985 (50.2)	10,135 (48.6)	2850 (60.2)	< 0.001
Alcohol drinkers	16,283 (68.2)	13,452 (69.6)	2831 (59.3)	< 0.001
*Education*				< 0.001
Below high school	8041 (19.5)	5955 (17.2)	2086 (33.7)	
High school	5937 (24.6)	4796 (24.1)	1141 (27.4)	
College or above	11,737 (55.8)	10,160 (58.6)	1577 (38.5)	
Missing	46 (0.1)	22 (0.1)	24 (0.4)	
Physically active	6798 (31.5)	5949 (33.3)	849 (19.9)	< 0.001
*Disease history*				
Hypertension	14,392 (49.8)	10,918 (46.6)	3474 (70.0)	< 0.001
Hyperlipidemia	15,929 (62.2)	12,972 (62.0)	2957 (63.3)	0.17
Diabetes	5636 (16.3)	4155 (14.5)	1481 (27.7)	< 0.001
CVD	4184 (13.1)	2499 (9.8)	1685 (34.2)	< 0.001
Cancer	3249 (13.1)	2209 (11.5)	1040 (23.0)	< 0.001
Neutrophils (× 10^9^/L)	3.97 (3.10–5.04)	3.93 (3.06–4.97)	4.26 (3.34–5.41)	< 0.001
Lymphocytes (× 10^9^/L)	1.89 (1.51–2.37)	1.91 (1.54–2.38)	1.74 (1.33–2.27)	0.19
Platelets (× 10^9^/L)	246 (208–291)	247 (209–292)	241 (199–289)	0.01
SII (× 10^9^/L)^c^	505.14 (366.96–705.63)	497.68 (363.66–690.55)	562.92 (392.70–824.09)	< 0.001

Values were presented as n (%) for categorical variables and median (interquartile range) for continuous variables.

*DFTJ* Dongfeng-Tongji cohort, *NHANES* National Health and Nutrition Examination Survey, *BMI* body mass index, *CVD* cardiovascular diseases, *SII* systemic immune-inflammation index.

^a^*P* values were calculated by the Chi-square test or Mann–Whitney *U* test.

^b^*P* values were calculated by Rao-Scott χ^2^ test for categorical variables, which is design adjusted version of Pearson χ^2^ test, and by variance estimation adjusting for sampling weights for continuous variables.

^c^For each subject, SII value = neutrophil count/lymphocyte count × platelet count.

### The associations of baseline SII with total and cause-specific mortality

**Figure 1:**
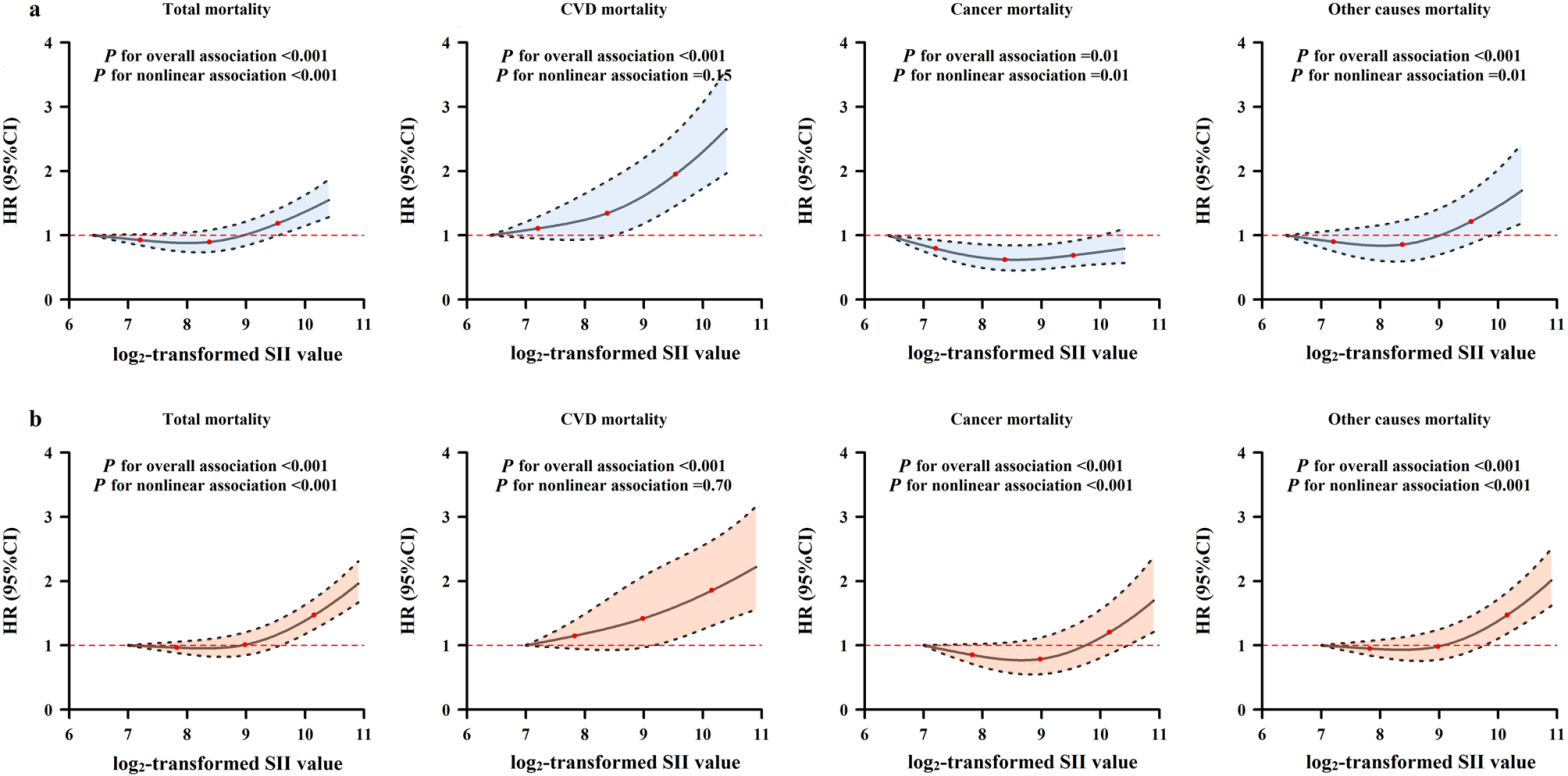
Estimated HRs (95%CIs) for the associations of SII level with risk of total and cause-specific mortality. (**a**) DFTJ cohort; (**b**) NHANES 1999–2014. *DFTJ* Dongfeng-tongji, *NHANES* National Health and Nutrition Examination Survey, *SII* systemic immune-inflammation index, *CVD* cardiovascular diseases. *Notes*: The HRs and 95% CIs were calculated by restricted cubic spline regression models, adjusted for age, sex, race (only in the NHANES 1999–2014), body mass index, smoking status, alcohol drinking status, physical activity status, education and baseline disease histories, with the minimum value of the transformed SII as reference (DFTJ cohort: 6.4; NHANES 1999–2014: 7.0). Knots were placed at the 5th, 50th and 95th percentiles of the SII.

In both cohorts, since the hazard ratios (HRs) for the associations of baseline SII with total and cause-specific mortality were relatively stable before the 50th percentile of SII and were steeper at a higher SII level (Fig. 1), we then classified participants into low, middle and high SII subgroups according to the 50th and 75th percentiles of SII among survivors in each cohort, and set the low SII subgroup as the reference. In the DFTJ cohort, subjects in the middle and high SII subgroups had higher risks of total mortality [HR (95%CI) = 1.12 (1.03–1.22) and 1.26 (1.16–1.36), respectively] and CVD mortality [HR (95%CI) = 1.36 (1.19–1.55) and 1.50 (1.32–1.71), respectively]; those in high SII subgroup had a significantly increased risk of other causes mortality [HR (95%CI) = 1.28 (1.09–1.49)]. There was no significant association between SII level and cancer mortality (*P* > 0.05). In the NHANES 1999–2014, subjects in the high SII subgroup had increased risks of total, CVD, cancer, and other causes mortality [HR (95%CI) = 1.38 (1.27–1.49), 1.33 (1.11–1.59), 1.22 (1.04–1.45) and 1.47 (1.32–1.63), respectively, Table 2]. In both cohorts, the sensitivity analyses by excluding subjects taking medicines that may influence the SII level, deaths occurring during the first year of follow-up, or subjects with prevalent CVD or cancer did not materially change the results (Supplementary Tables S2 and S3).

**Table 2:** The associations between the level of the systemic immune-inflammation index (SII) and mortality in the cohort studies.

SII category	Total mortality		CVD mortality		Cancer mortality		Other causes mortality	
	No of deaths/PYs	HR (95%CI)^a^	No of deaths	HR (95%CI)^a^	No of deaths	HR (95%CI)^a^	No of deaths	HR (95%CI)^a^
**DFTJ cohort**								
Low SII (< 332)	1580/127,963	Ref	578	Ref	577	Ref	425	Ref
Middle SII (332–465)	799/61,150	1.12 (1.03–1.22)	353	1.36 (1.19–1.55)	231	0.87 (0.75–1.02)	215	1.14 (0.97–1.34)
High SII (> 465)	932/59,909	1.26 (1.16–1.36)	408	1.50 (1.32–1.71)	273	1.00 (0.87–1.16)	251	1.28 (1.09–1.49)
**NHANES 1999–2014**								
Low SII (< 498)	2058/98,504	Ref	456	Ref	476	Ref	1126	Ref
Middle SII (498–691)	1098/47,705	1.07 (0.98–1.17)	266	1.03 (0.85–1.26)	218	0.97 (0.79–1.20)	614	1.13 (0.99–1.28)
High SII (> 691)	1672/49,564	1.38 (1.27–1.49)	371	1.33 (1.11–1.59)	338	1.22 (1.04–1.45)	963	1.47 (1.32–1.63)

*DFTJ* Dongfeng-Tongji cohort, *NHANES* National Health and Nutrition Examination Survey, *PY* person-year.

^a^HR (95%CI)s were calculated in the multivariable Cox proportional hazards regression models, with adjustment for age, sex, race (only in the NHANES 1999–2014), body mass index, smoking status, alcohol drinking status, education, physical activity status, and disease histories at baseline.

### Effect modifications by general characteristics and lifestyles

In the NHANES 1999–2014, we observed significant modification effect of physical activity on the association between SII and total mortality risk (*P*_int_ = 0.04, Supplementary Table S4). When assessing the cause-specific mortality, we found significant and marginal significant interaction effects between physical activity and SII on CVD and other causes mortality (*P*_int_ = 0.04 and 0.06, respectively), but not for cancer mortality (*P*_int_ = 0.47, Supplementary Table S5). However, we did not observe the significant interaction between physical activity and baseline SII in the DFTJ cohort, and non-significant interactions were found for age, sex, BMI, smoking and alcohol drinking status with SII in both cohorts (all *P*_int_ > 0.05, Supplementary Table S4).

We further assessed the effect of physical activity on mortality risk across SII subgroups in both cohorts. As shown in Table 3, in the DFTJ cohort, physical activity could significantly attenuate total mortality risk by 29–39% across SII subgroups, and corresponding HRs (95%CIs) in low, middle and high SII subgroups were 0.71 (0.63–0.80), 0.61 (0.52–0.72) and 0.70 (0.60–0.81), respectively. Similar effects of physical activity were observed for CVD mortality and other causes mortality, with risk reduction by 31–41% and 35–42%, respectively. Physical activity decreased cancer mortality by 37% in the middle SII group, but such effect was not shown in low or high SII subgroups. In the NHANES 1999–2014, physical activity was also found to be associated with lower risk of total mortality across low, middle and high SII subgroups [HR (95%CI) = 0.69 (0.60–0.80), 0.65 (0.52–0.80) and 0.59 (0.51–0.68), respectively]. Physical activity was only significantly associated with 59% reduced CVD mortality in the high SII subgroup [HR (95%CI) = 0.41 (0.27–0.64)], and 36% lower cancer mortality in the low SII subgroup [HR (95%CI) = 0.64 (0.47–0.88)].

**Table 3:** The association between physical activity and mortality risk stratified by SII groups.

Physical activity status	Low SII	Middle SII	High SII
**DFTJ cohort**			
*Total mortality*			
Inactive	Ref	Ref	Ref
Active	0.71 (0.63–0.80)	0.61 (0.52–0.72)	0.70 (0.60–0.81)
*CVD mortality*			
Inactive	Ref	Ref	Ref
Active	0.69 (0.57–0.84)	0.59 (0.46–0.75)	0.68 (0.54–0.85)
*Cancer mortality*			
Inactive	Ref	Ref	Ref
Active	0.88 (0.71–1.09)	0.63 (0.46–0.85)	0.83 (0.62–1.12)
*Other causes mortality*			
Inactive	Ref	Ref	Ref
Active	0.58 (0.46–0.72)	0.65 (0.47–0.90)	0.62 (0.47–0.83)
**NHANES 1999–2014**			
*Total mortality*			
Inactive	Ref	Ref	Ref
Active	0.69 (0.60–0.80)	0.65 (0.52–0.80)	0.59 (0.51–0.68)
*CVD mortality*			
Inactive	Ref	Ref	Ref
Active	0.76 (0.55–1.03)	0.78 (0.50–1.20)	0.41 (0.27–0.64)
*Cancer mortality*			
Inactive	Ref	Ref	Ref
Active	0.64 (0.47–0.88)	0.71 (0.49–1.04)	0.86 (0.62–1.19)
*Other causes mortality*			
Inactive	Ref	Ref	Ref
Active	0.69 (0.58–0.83)	0.58 (0.45–0.75)	0.57 (0.46–0.70)

HR (95%CI)s were calculated in the multivariable Cox proportional hazards regression models, with adjustment for age, sex, race (only in the NHANES 1999–2014), body mass index, smoking status, alcohol drinking status, education, and disease histories.

*DFTJ* Dongfeng-Tongji cohort, *NHANES* National Health and Nutrition Examination Survey, *SII* systemic immune-inflammation index.

## Discussion

To our knowledge, this is the first study using two large prospective cohorts from China and U.S. to explore the association between SII level and total and cause-specific mortality among the general middle-aged and older populations. The results demonstrated that the high SII was associated with a separate 26% and 50% increased risk of total and CVD mortality in the DFTJ cohort, as well as a separate 38% and 33% increased risk of total and CVD mortality in the NHANES 1999–2014. Moreover, physical activity could reduce a separate 30% and 32% risk of total and CVD mortality in the high SII subgroup of the DFTJ cohort, and decrease a separate 41% and 59% risk of total and CVD mortality in the high SII subgroup of the NHANES 1999–2014.

Multiple inflammatory biomarkers have been studied on their associations with risk of CVD, cancer and mortality individually^10,24,25^. Morrison L. suggested that combining inflammatory markers into a risk score was a robust method for testing the associations of systemic chronic inflammation with cancer risk and cancer mortality^26^. The SII is an addition to these combined inflammatory makers, which integrates different but complementary pathways simultaneously. The low cost and common availability of blood routine test in primary care make the SII an attractive index for further investigation.

Many studies have indicated that a high level of SII was associated with early recurrence and poor survival among certain cancer patients, and these effects were independent of conventional risk factors and other inflammatory scores or markers^17,27–29^. Besides the investigations in the clinical patients, two studies suggested that individuals with high SII had elevated risks of solid cancer and dementia in the general population^20,21^. In the present study, we observed significant associations between high SII and increased total and CVD mortality in middle-aged and older populations. The effect of high SII on elevated total mortality was consistent with the findings reported by a previous study of 85,154 Chinese participants^22^. It was shown that high SII was associated with increased cancer mortality for subjects in NHANES 1999–2014, but this association was not observed in the DFTJ cohort, possibly due to the different combinations of cancer subtypes between the two cohorts. However, we could not further evaluate the associations of SII level with the mortality of specific type of cancer because of the limited information of cancer subtypes of deaths.

High level of SII was reported to be associated with elevated cytokines, e.g., interleukin-6 (IL-6), IL-8, IL-10^30,31^, which were implicated in the systemic chronic inflammation response. The increased mortality in participants with higher SII level was thought to be driven by chronic inflammation, which could generate reactive nitrogen species, reactive oxygen species, genomic instability and cell senescence, increasing the risk of CVD and mortality^1^. Moreover, SII was reported to be associated with circulating tumor cells in hepatocellular carcinoma patients^17^. The interactions between the circulating neutrophils and platelets help cancer cells evade immune surveillance and eradication. It was suggested that the high SII level may reflect the suppression of immune function, which supports proliferation, invasion and metastasis of cancer cells.

Previous epidemiological studies have reported the benefic effects of physical activity on mortality risk^32,33^. In the current study, physical activity attenuated total and CVD mortality significantly, especially in subjects with high level of SII. Similar with our results, a cohort study including 336,560 South Korean participants found that physical activity could attenuate the association between high-sensitivity C-reactive protein and CVD mortality, and could also reduce the risks of total and cause-specific mortality among those with high high-sensitivity C-reactive protein^34^. These findings emphasize the importance of physical activity on the prevention of total and CVD mortality related to chronic inflammation. Although the protective effect of physical activity against cancer mortality did not consistently reach the significant level in each subgroup of SII, several epidemiological studies reported inverse associations of physical activity with cancer mortality^32,33^. An umbrella review summarizing 19 systematic reviews on the associations of physical activity with cancer incidence and cancer mortality suggested strong associations with breast and colon cancers, but evidence for other cancer sites was less consistent and uncertain^35^. These findings emphasized the need of further explorations on specific types of cancers.

The present study involved two large sample sized cohort studies from China and U.S., which could ensure the statistical power and causal inference for the current findings. The generally consistent results among populations from two countries make the findings robust and reliable. However, some limitations should be noted. First, the one-spot blood routine measurement and SII at baseline might not represent a long-time status of chronic inflammation across the follow-up period and lead to underestimation of the association between SII and mortality. However, a previous study with two measurements over 6.1 years showed that the within-person change of the SII was small^36^. Second, most of the participants in the DFTJ cohort were elder retirees, which limited representativeness for the younger Chinese population. However, the general characteristics and SII level among the DFTJ cohort participants were comparable with that reported in the general middle-aged and older Chinese population^37,38^. Thirdly, our results might be affected by reverse causation or residual confounding. However, the associations remained even after excluding subjects who died during the first year of follow-up or subjects with prevalent CVD and cancer, which minimize the possibility of reverse causation. Fourthly, lifestyle factors were defined differently in two studies. However, when stratified by these factors, the associations of SII with mortality in each stratum were similar in both studies, which suggested the findings were not explainable by chance. Finally, the observational study could not provide mechanistic information, therefore the biology underlying the results is unknown. Further in vivo and in vitro studies are also warranted to clarify the possible mechanisms.

## Conclusions

Our studies found that high level of SII was associated with a separate 26–38% and 33–50% increased risk of total and CVD mortality in the DFTJ cohort and NHANES 1999–2014. For subjects with a high level of SII, physical activity could attenuate a separate 30% and 32% risks of total and CVD mortality in the DFTJ cohort, and a separate 41% and 59% risk of total and CVD mortality in the NHANES 1999–2014. In a public health view, regular physical exercise was suggested as a promising strategy to prevent the chronic inflammation caused elevated risk of mortality in the general middle-age and older populations.

## Methods

### Study populations

The study designs of the DFTJ cohort and NHANES have been described previously^39,40^. Briefly, the DFTJ cohort began in 2008, launched among retirees from Dongfeng Motor Corporation in China, and was followed up every five years. A total of 27,009 participants were recruited at baseline and additional 14,120 retirees were recruited during the first follow-up visit in 2013. The NHANES was conducted periodically from 1999 and selected about 5000 persons annually in counties across the United States (U.S.) using complex, multistage probability sampling design to represent the general U.S. population of all ages. These data were publicly released in 2-year cycles. In the current study, we combined 8 consecutive survey cycles during 1999–2014 with a total of 82,091 participants (response rate ranged 71.0–83.9% in 8 cycles).

**Figure 2:**
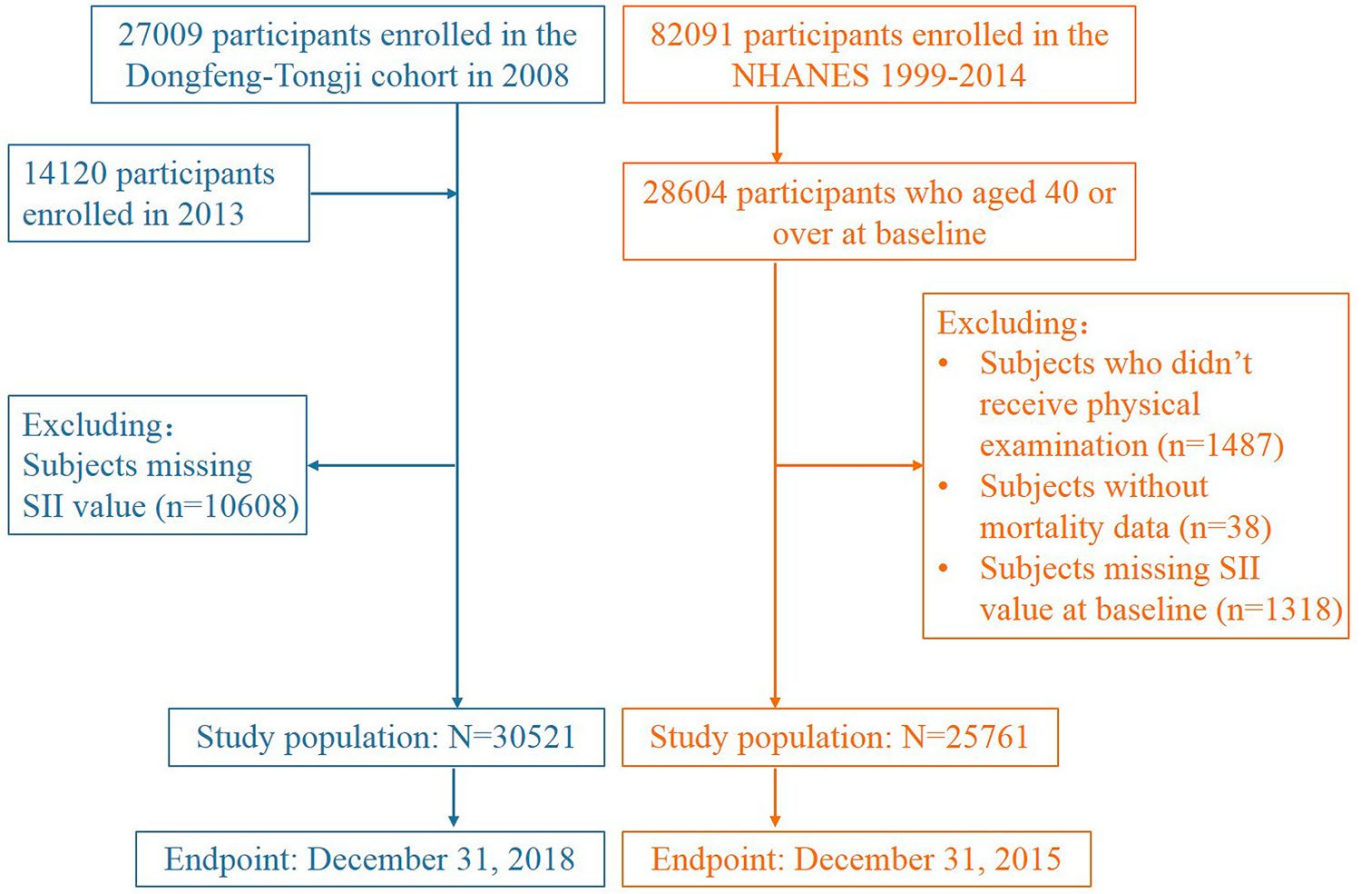
Flow chart of study participants.

In the DFTJ cohort, we excluded individuals without value of SII (n = 10,608 without information of neutrophil, lymphocyte, or platelet counts). Therefore, the left 30,521 participants were included in this study. In the NHANES 1999–2014, after excluding individuals who did not take health examination (n = 1487), without mortality data (n = 38), and without information of related blood cells count (n = 1318), a total of 25,761 participants aged ≥ 40 years old were included because most deaths occurred in these adults (Fig. 2).

### Assessment of covariates

Information on demographic characteristics (e.g., age, sex, race, and education level), lifestyles (e.g., smoking status, alcohol drinking status, and physical activity status), medical history, and self-reported diseases was assessed with a self-administered questionnaire and an interview during the baseline data collection. The body weight and height were measured in the physical examination. In both studies, BMI was calculated as weight divided by the square of height (kg/m^2^). In the questionnaires of DFTJ cohort, current smokers were defined as participants who have smoked ≥ 1 cigarette/day in the past half a year, former smokers as participants who had ever smoked but stopped for at least half a year, and never smokers as participants who have smoked < 1 cigarette/day in lifetime. Current alcohol drinkers were defined as participants who have ≥ 1 drink/week in the past half a year, former drinkers as participants who had ever drunk but stopped for at least half a year, and never drinkers as participants who have < 1 drink/week in lifetime. Current and former smokers or drinkers were combined as smokers or drinkers. Participants were asked whether they did physical activity for more than 20 min each time in leisure time during the past half a year, and those with an affirmative answer were then asked total frequency per week and duration each time. Total weekly physical activity duration was calculated by frequency per week multiplying the average minutes each time.

In the NHANES 1999–2014, smokers were defined as participants who had smoked ≥ 100 cigarettes during lifetime, otherwise, they were non-smokers. Alcohol drinkers were defined as participants who had ≥ 12 drinks in any 1 year, otherwise, they were non-drinkers. Participants were asked whether did any vigorous-intensity recreational activities, as well as whether they did any moderate-intensity recreational activities for at least 10 min continuously. Those with an affirmative response to either question were also asked the frequency and duration each day of the specific moderate or vigorous-intensity activities. Weekly duration of moderate or vigorous-intensity activities was calculated as frequency per week multiplying minutes each day. To make the same classification criteria of physical activity status in both cohorts, total physical activity duration per week was calculated as the sum of weekly minutes of vigorous-intensity activities and weekly minutes of moderate-intensity activities. In both cohorts, participants with total physical activity ≥ 150 min/week were classified as physically active, otherwise, they were physically inactive^41^.

### Measurement of the peripheral counts of blood cells

The peripheral counts of blood cells were measured by certified technologists using CELL-DYN 3700 system (Abbott Laboratories, Illinois, USA) in the DFTJ cohort and Beckman Coulter Counter (Beckman Coulter Inc., Brea, CA, USA) in the NHANES 1999–2014. The SII level was calculated for each participant as follows: SII (× 10^9^/L) = neutrophil count (× 10^9^/L)/lymphocyte count (× 10^9^/L) × platelet count (× 10^9^/L)^17^.

### Mortality follow-up

In the DFTJ cohort, each subject had a unique medical insurance number to track the vital status through Dongfeng Motor Corporation’s medical insurance system, which covers all retirees. The causes of death for the dead were confirmed by medical records from hospitals and death certificates from Centers for Disease Control and Prevention. This information was verified mutually. For the participants in the NHANES 1999–2014, the public-use linked mortality files were released by the National Center for Health Statistics, including vital status, cause of death, follow-up time from interview date and follow-up time from physical examination date. Mortality variables were obtained by matching to public-use linked mortality files using exclusive respondent sequence number^42^. Mortality data was available till December 31, 2018 in the DFTJ cohort and December 31, 2015 in the NHANES 1999–2014. We used the 10th revision of the international classification of diseases (ICD-10) to classify the causes of death. Total and cause-specific mortality, including CVD mortality (codes I00-I09, I11-I13, I20-I51, I60-I69), cancer mortality (codes C00-C97), and other causes mortality were assessed.

### Statistical analysis

Data were reported as median (IQR) for continuous variables, with differences tested by Mann–Whitney *U* test, or number (percentage) for categorical variables, with different distributions tested by χ^2^ test. SII value was log_2_-transformed to conform to normal distribution. Multivariable linear regression was conducted to disclose the associations of SII level with basic characteristics, including age, sex, race (only in the NHANES 1999–2014), BMI, smoking status, alcohol drinking status, and physical activity status. We fitted restricted cubic spline models to show the dose–response association shapes of SII with mortality, with 3 knots at the 5th, 50th and 95th percentiles of the SII. Subjects with extreme SII value outside their 1st and 99th percentiles were excluded due to limited number of participants and events. The hazard ratios (HRs) for the associations of SII with total and cause-specific mortality were relatively stable before the 50th percentile of SII and were steeper at a higher SII level, therefore, we classified participants into three subgroups: low SII: < 50th percentile; middle SII: 50th–75th percentile; and high SII: > 75th percentile of SII among survivors in each cohort. HRs and 95% CIs were calculated to examine the associations of SII subgroups with mortality by using Cox proportional hazards regression models, with adjustment for baseline age (continuous), sex, race (only in the NHANES 1999–2014), BMI (< 18.5, 18.5–24.9, 25.0–29.9, ≥ 30 kg/m^2^), education level (below high school, high school, college or above), smoking status (smokers, non-smokers), alcohol drinking status (drinkers, non-drinkers), physical activity status (physically active, physically inactive), and baseline disease histories of hypertension, hyperlipidemia, diabetes, CVD and cancer. Person-years were recorded from baseline interview to date of death, loss to follow-up, or end of follow-up, whichever came first. The proportional hazard assumption was tested by adding cross term between person-years and exposure factors, showing no violations. Several sensitivity analyses were further conducted by excluding: (1) subjects taking anti-coagulation drugs, thrombolytic drugs, antibiotics and aspirin, which may influence SII level at baseline; (2) subjects who died during the first year of follow-up; (3) participants with prevalent CVD or cancer.

The stratified analyses were conducted by sex, age, BMI, smoking status, alcohol drinking status, and physical activity status. Effect modification was evaluated by likelihood ratio tests to compare the models with or without the interaction term between each of these covariates and SII. We further explored the beneficial effect of physical activity on mortality risk in each SII subgroup.

Sample weights, clustering, and stratification were taken into account for the complex sampling design in all analyses among the NHANES 1999–2014. All statistical analyses were performed by using SAS 9.4 (SAS Institute Inc. Cary, NC, USA), and two-tailed *P* < 0.05 were considered statistically significant.

### Ethical approval

Written informed consents were obtained from all participants. The DFTJ cohort was approved by the Ethics and Human Subject Committee of the School of Public Health, Tongji Medical College, Huazhong University of Science and Technology; the NHANES was approved by the National Center for Health Statistics Research Ethics Review Board. The methods were conducted in accordance with the approved guidelines and regulations.

## Supplementary Information

Supplementary Information.

## Data Availability

The dataset of DFTJ cohort is available from the corresponding author upon reasonable request and the dataset of the NHANES is available at https://www.cdc.gov/nchs/nhanes/.

## Abbreviations

CVD: Cardiovascular disease
WBC: White blood cell
NLR: Neutrophil-to-lymphocyte ratio
PLR: Platelet-to-lymphocyte ratio
SII: Systemic immune-inflammation index
DFTJ: Dongfeng-Tongji
NHANES: National Health and Nutrition Examination Survey
BMI: Body mass index
ICD-10: 10th Revision of the international classification of diseases
IQR: Interquartile range
HR: Hazard ratio
CI: Confidence interval
IL: Interleukin
