# Supplementary material for: Physical activity attenuates the associations of systemic immune-inflammation index with total and cause-specific mortality among middle-aged and older populations
Source: Sci Rep. 2021 Jun 15;11:12532. doi: 10.1038/s41598-021-91324-x (PMC8206152; doi:10.1038/s41598-021-91324-x)
Supplement: Supplementary file 1 — Supplementary Information. [file 41598_2021_91324_MOESM1_ESM.pdf]

Physical activity attenuates the associations of systemic immune-inflammation index with total and cause-specific mortality among middle-aged and older populations

Hang Li, Xiulong Wu, Yansen Bai, Wei Wei, Guyanan Li, Ming Fu, Jiali Jie, Chenming Wang, Xin Guan, Yue Feng, Hua Meng, Mengying Li, Meian He, Xiaomin Zhang, Huan Guo*

Supplementary Tables

Table S1. The associations between baseline characteristics and SII level.

Table S2. Sensitivity analyses for the associations between the level of SII and mortality risks among the Dongfeng-Tongji cohort.

Table S3. Sensitivity analyses for the associations between the level of SII and mortality risks among the NHANES 1999-2014.

Table S4. The associations between SII and total mortality in subgroups of cohort participants.

Table S5. The associations of SII with cause-specific mortality stratified by physical activity in the NHANES 1999-2014.

Table S1. The associations between baseline characteristics and SII level.

Variables	DFTJ cohort	NHANES 1999-2014
Age	-0.008 (-0.010, -0.007)	-0.001 (-0.002, -0.0003)
Male	-0.019 (-0.043, 0.006)	-0.094 (-0.115, -0.072)
Race		
Non-Hispanic White	-	Ref
Non-Hispanic Black	-	-0.442 (-0.476, -0.409)
Mexican American	-	-0.102 (-0.133, -0.070)
Other	-	-0.144 (-0.188, -0.101)
BMI	0.0004 (-0.002, 0.003)	0.005 (0.003, 0.007)
Smoking	0.116 (0.091, 0.142)	0.053 (0.024, 0.083)
Alcohol drinking	-0.017 (-0.039, 0.006)	-0.013 (-0.042, 0.017)
Physically active	-0.035 (-0.058, -0.013)	-0.090 (-0.114, -0.066)

Abbreviations: SII, systemic immune-inflammation index; DFTJ, Dongfeng-Tongji ; NHANES, National Health and Nutrition Examination Survey; BMI, body mass index.
Note: β (95%CI)s were calculated by multivariable linear regression models, with adjustment of age, sex, race (only in the NHANES 1999-2014), BMI, smoking status, alcohol drinking status, physical activity and disease history at baseline.

Table S2. Sensitivity analyses for the associations between the level of SII and mortality risks among the Dongfeng-Tongji cohort.

SII category	Total mortality		CVD mortality		Cancer mortality		Other causes mortality	
	**No of deaths/PYs**	**HR (95%CI) [a]**	**No of deaths**	**HR (95%CI) [a]**	**No of deaths**	**HR (95%CI) [a]**	**No of deaths**	**HR (95%CI) [a]**
Excluded subjects who took drugs two weeks before baseline [b] (n=6755)								
Low SII (<332)	1137/100052	Ref	384	Ref	446	Ref	307	Ref
Middle SII (332-465)	569/47563	1.12 (1.01-1.24)	240	1.41 (1.20-1.66)	180	0.88 (0.74-1.05)	149	1.10 (0.91-1.34)
High SII (>465)	672/471119	1.25 (1.14-1.38)	291	1.60 (1.38-1.87)	209	0.99 (0.84-1.17)	172	1.22 (1.01-1.47)
Excluded subjects who died during the first year of follow-up (n=138)								
Low SII (<332)	1516/127921	Ref	555	Ref	557	Ref	404	Ref
Middle SII (332-465)	766/61131	1.13 (1.03-1.23)	335	1.35 (1.18-1.55)	226	0.88 (0.76-1.03)	205	1.15 (0.97-1.36)
High SII (>465)	891/59882	1.26 (1.16-1.37)	393	1.52 (1.33-1.73)	254	0.97 (0.83-1.13)	244	1.32 (1.12-1.55)
Excluded subjects with baseline history of CVD and cancer (n=5915)								
Low SII (<332)	1011/103778	Ref	351	Ref	382	Ref	278	Ref
Middle SII (332-465)	521/49762	1.11 (1.00-1.23)	215	1.32 (1.11-1.56)	157	0.88 (0.73-1.07)	149	1.16 (0.95-1.41)
High SII (>465)	570/48368	1.22 (1.10-1.36)	245	1.51 (1.29-1.79)	168	0.96 (0.80-1.15)	157	1.23 (1.01-1.49)

Abbreviations: SII, systemic immune-inflammation index; CVD, cardiovascular disease; PY, person year.
Note: [a] HR (95%CI)s were calculated in the multivariable Cox proportional hazards regression models, with adjustment for age, sex, body mass index, smoking status, alcohol drinking status, physical activity, education, and disease histories.
[b] Drugs included anti-coagulation drugs, thrombolytic drugs, antibiotics and aspirin.

Table S3. Sensitivity analyses for the associations between the level of SII and mortality risks among the NHANES 1999-2014.

SII category	Total mortality		CVD mortality		Cancer mortality		Other causes mortality	
	No of deaths/PYs	HR (95%CI) [a]	No of deaths	HR (95%CI) [a]	No of deaths	HR (95%CI) [a]	No of deaths	HR (95%CI) [a]
Excluded subjects who took drugs two weeks before baseline [b] (n=3198)								
Low SII (<498)	1665/88858	Ref	363	Ref	396	Ref	906	Ref
Middle SII (498-691)	879/42847	1.09 (0.99-1.20)	206	1.00 (0.80-1.25)	184	1.03 (0.82-1.29)	489	1.16 (1.00-1.34)
High SII (>691)	1253/42785	1.38 (1.26-1.52)	268	1.26 (1.02-1.55)	279	1.35 (1.12-1.62)	706	1.45 (1.29-1.63)
Excluded subjects who died during the first year of follow-up (n=377)								
Low SII (<498)	1919/98416	Ref	411	Ref	449	Ref	1059	Ref
Middle SII (498-691)	1038/47663	1.07 (0.98-1.17)	247	1.08 (0.87-1.32)	205	0.95 (0.77-1.18)	586	1.12 (0.98-1.28)
High SII (>691)	1507/49459	1.33 (1.22-1.45)	331	1.39 (1.15-1.67)	299	1.15 (0.97-1.37)	877	1.40 (1.25-1.56)
Excluded subjects with baseline history of CVD and cancer (n=6579)								
Low SII (<498)	1167/77816	Ref	231	Ref	296	Ref	640	Ref
Middle SII (498-691)	571/37260	1.05 (0.92-1.20)	130	1.13 (0.84-1.52)	114	0.86 (0.62-1.20)	327	1.13 (0.93-1.36)
High SII (>691)	776/36555	1.29 (1.16-1.43)	153	1.38 (1.06-1.78)	152	1.04 (0.81-1.33)	471	1.38 (1.22-1.57)

Abbreviations: NHANES, National Health and Nutrition Examination Survey; SII, systemic immune-inflammation index; CVD, cardiovascular disease; PY, person year.

[a] HR (95%CI)s were calculated in the multivariable Cox proportional hazards regression models, with adjustment for age, sex, race, body mass index, smoking status, alcohol drinking status, physical activity, education, and disease histories.

[b] Drugs included anti-coagulation drugs, antibiotics and aspirin.

Table S4. The association between SII level and total mortality in subgroups of cohort participants.

**Characteristics**	Low SII	Middle SII	High SII	P_{int}
DFTJ cohort				
Sex				0.37
Male	Ref	1.17 (1.05-1.30)	1.29 (1.16-1.42)	
Female	Ref	1.03 (0.89-1.19)	1.19 (1.04-1.37)	
Age				0.56
<65 years	Ref	1.00 (0.86-1.17)	1.15 (0.99-1.33)	
≥65 years	Ref	1.12 (1.02-1.24)	1.27 (1.15-1.39)	
BMI				0.09
<25 kg/m^2	Ref	1.07 (0.95-1.20)	1.30 (1.17-1.44)	
≥25 kg/m^2	Ref	1.21 (1.07-1.38)	1.20 (1.05-1.37)	
Smoking status				0.25
Non-smokers	Ref	1.13 (1.01-1.27)	1.19 (1.06-1.34)	
Smokers	Ref	1.12 (0.99-1.28)	1.34 (1.19-1.51)	
Drinking status				0.53
Non-drinkers	Ref	1.14 (1.03-1.27)	1.31 (1.18-1.44)	
Drinkers	Ref	1.08 (0.93-1.25)	1.17 (1.01-1.34)	
Physical activity status				0.36
Inactive	Ref	1.24 (1.04-1.48)	1.25 (1.05-1.48)	
Active	Ref	1.09 (0.99-1.21)	1.27 (1.15-1.39)	
NHANES 1999-2014				
Sex				0.31
Male	Ref	1.07 (0.93-1.23)	1.31 (1.16-1.47)	
Female	Ref	1.06 (0.94-1.21)	1.43 (1.26-1.63)	
Age				0.07
<65 years	Ref	1.02 (0.83-1.26)	1.27 (1.08-1.50)	
≥65 years	Ref	1.11 (1.01-1.21)	1.45 (1.34-1.57)	
BMI				0.90
<25 kg/m^2	Ref	1.04 (0.89-1.22)	1.35 (1.15-1.59)	
≥25 kg/m^2	Ref	1.05 (0.94-1.16)	1.38 (1.25-1.54)	
Smoking status				0.28
Non-smokers	Ref	0.97 (0.85-1.11)	1.29 (1.14-1.46)	
Smokers	Ref	1.14 (0.99-1.30)	1.44 (1.27-1.62)	
Drinking status				0.19
Non-drinkers	Ref	1.07 (0.93-1.24)	1.44 (1.25-1.67)	
Drinkers	Ref	1.08 (0.95-1.23)	1.32 (1.18-1.48)	
Physical activity status				0.04
Inactive	Ref	1.08 (0.97-1.21)	1.43 (1.29-1.58)	
Active	Ref	1.00 (0.78-1.29)	1.14 (0.96-1.37)	

Abbreviations: DFTJ, Dongfeng-Tongji cohort; NHANES, National Health and Nutrition Examination Survey; SII, systemic immune-inflammation index; BMI, body mass index. Note: HR(95%CI)s were calculated in the multivariable Cox proportional hazards regression models , with adjustment for age, sex, race (only in the NHANES 1999-2014), BMI, smoking status, alcohol drinking status, physical activity, education, and disease histories, except for the stratification variable.

Table S5. The associations of SII level with cause-specific mortality stratified by physical activity in the NHANES 1999-2014.

**Physical activity status**	**Low SII**	**Middle SII**	**High SII**	P_{int}
**CVD mortality**				**0.04**
Inactive	Ref	1.03 (0.85-1.24)	1.44 (1.18-1.77)	
Active	Ref	1.01 (0.60-1.69)	0.83 (0.51-1.35)	
**Cancer mortality**				0.47
Inactive	Ref	0.95 (0.74-1.22)	1.18 (0.99-1.41)	
Active	Ref	1.02 (0.68-1.55)	1.35 (0.92-1.99)	
**Other causes mortality**				0.06
Inactive	Ref	1.16 (1.00-1.35)	1.54 (1.36-1.74)	
Active	Ref	0.99 (0.73-1.33)	1.17 (0.91-1.49)	

Abbreviations: DFTJ, Dongfeng-Tongji cohort; NHANES, National Health and Nutrition Examination Survey; SII, systemic immune-inflammation index.
Note: HR (95%CI)s were calculated in the multivariable Cox proportional hazards regression models, with adjustment for age, sex, race (only in the NHANES 1999-2014), body mass index, smoking status, alcohol drinking status, education, and disease histories.
